# Interaction of Reactive-Dye Chromophores and DEG on Ink-Jet Printing Performance

**DOI:** 10.3390/molecules25112507

**Published:** 2020-05-28

**Authors:** Liyuan Zhang, Kuanjun Fang, Hua Zhou

**Affiliations:** 1 College of Textiles & Clothing, Qingdao University, 308 Ningxia Road, Qingdao 266071, China; zliy1997@163.com; 2 State Key Laboratory for Biofibers and Eco-textiles, 308 Ningxia Road, Qingdao 266071, China; 3 Collaborative Innovation Center for Eco-textiles of Shandong Province, 308 Ningxia Road, Qingdao 266071, China

**Keywords:** ink-jet printing, diethylene glycol, reactive dye, dye chromophores, printing performance

## Abstract

Digital inkjet printing has been widely used in textile industry. The quality of dye solutions and ink-jet droplets limits the ink-jet printing performance, which is very important for obtaining high-quality ink-jet printing images on fabrics. In this paper, we introduced diethylene glycol (DEG) into the dye solutions of Reactive Blue 49 and Reactive Orange 13, respectively, and investigated the interaction between dye chromophores and DEG molecules. Results indicated that the dye chromophores were featured in the aggregation. Adding DEG into the dye solution could effectively disaggregate clusters of reactive dyes, and eliminate satellite ink droplets, thus improving the resolution of the ink-jet printing image on fabrics. Under the same DEG concentration, the disaggregation effect was more obvious in Orange 13 than in Reactive Blue 49. Higher DEG concentration was required in Reactive Orange 13 solution for creating complete and stable ink drops. The surface tension and viscosity of the dye solutions were measured, and printing performance on cotton fabrics was evaluated. The interaction mechanism between dye chromophores and DEG molecules was also investigated. Results from this work are useful for high-quality ink-jet printing images on fabrics.

## 1. Introduction

Digital ink-jet printing technology has been applied in many fields because of its incomparable advantages, such as low cost, facility, celerity, and ecofriendliness [1,2,3,4,5,6,7,8,9,10]. Reactive dyes are widely used for cotton-fabric dyeing in ink-jet printing techniques due to their bright color, excellent performance, and good applicability [2,11,12,13,14,15]. However, the relative coarse resolution of printed images is still a limitation of this technology. First, reactive-dye molecules are prone to aggregating in an aqueous solution through intermolecular forces such as hydrogen bonding and van der Waals forces [16,17]. More aggregation can be found in deep-color dyeing, while highly concentrated dye concentration is needed. Too much aggregation may result in dye crystallization and precipitation, which leads to nozzle blockage during the ink-jet printing process [18,19]. This phenomenon is a major obstacle for ink-jet printing uniform images. Much attention has been paid to suppressing the formation of aggregation through the understanding of the physical and chemical properties of reactive dyes, and the interactions of dyes and additives [16,17,19,20,21,22,23,24]. Second, poor ink-jet droplet formation, such as satellite droplets, is also a serious problem that reduces the resolution of printed images [25,26,27]. During the ink-jet printing process, solutes/inks prefer to deposit along the droplet periphery and form low-resolution images with inhomogeneous morphology and unclear borders on the fabric [6,25,26,27].

In our previous work, we compared four different reactive dyes, and investigated the interaction between reactive-dye molecules and surface tension, and the viscosity of the dye solutions [28]. The hydrophobic groups in reactive-dye molecules can decrease surface tension and increase the viscosity of the dye solution. However, the relationship between dye molecules and solvents is not yet clear.

**Figure 1:**
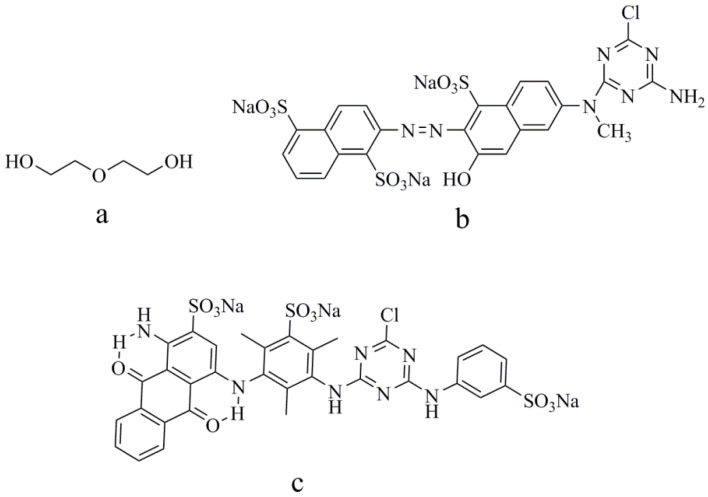
Chemical structures of: (**a**) diethylene glycol (DEG), (**b**) Reactive Orange 13, and (**c**) Reactive Blue 49.

Alcohol water-based solvent has become one of the most commonly used solvents due to its nontoxic environmentally friendly properties. In this paper, diethylene glycol (DEG) was applied as an organic solvent to prepare reactive-dye solutions/inks. Reactive Blue 49 with anthraquinone structure and Reactive Orange 13 with azo structure were selected as the reactive-dye models. The chemical structures of Blue 49, Orange 13, and diethylene glycols are shown in Figure 1. The effects of reactive-dye chromophores on jetting performance and the printed images were investigated. Results showed that reactive-dye chromophores were featured in the aggregation behavior in the aqueous solutions. Adding DEG into the dye solutions can not only effectively suppress the formation of dye-molecule aggregation in the solutions, but also decrease the surface tension, thus improving the droplet formation. At the optimal condition, used DEG concentrations in Reactive Orange 13 and Reactive Blue 49 solutions are different. A higher DEG concentration is required in the Reactive Orange 13 dye solution to eliminate satellite ink droplets and create ideal droplets. Under the same DEG concentration, the disaggregation effect was more obvious in Reactive Orange 13 than in that of Reactive Blue 49. UV–visible absorption spectra was used to study the aggregation behavior of dye molecules in aqueous solutions. The surface tension and viscosity of dye solutions were measured. Droplet formation and ink-jet printing performance on cotton fabrics were evaluated.

## 2. Results and Discussion

### 2.1. Interaction between DEG and Dyes

The aggregation state of inks is an important factor affecting their inkjet-printing behaviors [24]; a large aggregation of dyes may lead to nozzle blockage and make dye molecules difficult to penetrate, and spread into fabrics. 

**Figure 2:**
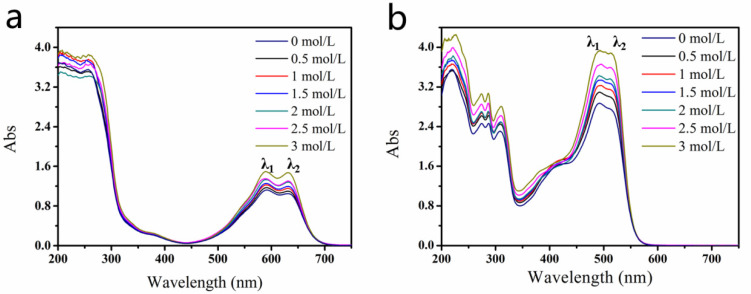
UV–visible absorption spectra of dye solutions with different DEG amounts. (**a**) Blue 49, (**b**) Orange 13 (dye concentration, 120 mmol/L).

The presence of aggregates in the dye solutions may influence their photophysical behavior, which can be detected from the shape of the absorption spectra in the visible region [29,30,31,32], so UV–Vis absorption spectroscopy is an important method for studies of the aggregation state [17,18,19,30,31,32,33,34]. Figure 2 shows the absorption spectra of the Blue 49 and Orange 13 dye solutions with different concentrations of DEG. For both dyes, the two main peaks in the visible region came from the dye monomer and aggregation absorption, respectively, while longer wavelength peak λ_2_ belonged to monomer absorption, and shorter peak λ_1_ came from aggregation absorption [33,35]. After adding DEG into the dye solutions, the two main peaks of both dye solutions increased, which meant that DEG molecules could separate large dye aggregations into more small aggregations and dye monomers. In comparison with Blue 49, Orange 13 had more obviously enhanced absorption peaks, which indicated that the same DEG concentration had more interaction with Orange 13 dye molecules, and may have facilitated the dye to releasing more small H-aggregates or even dye monomers.

**Figure 3:**
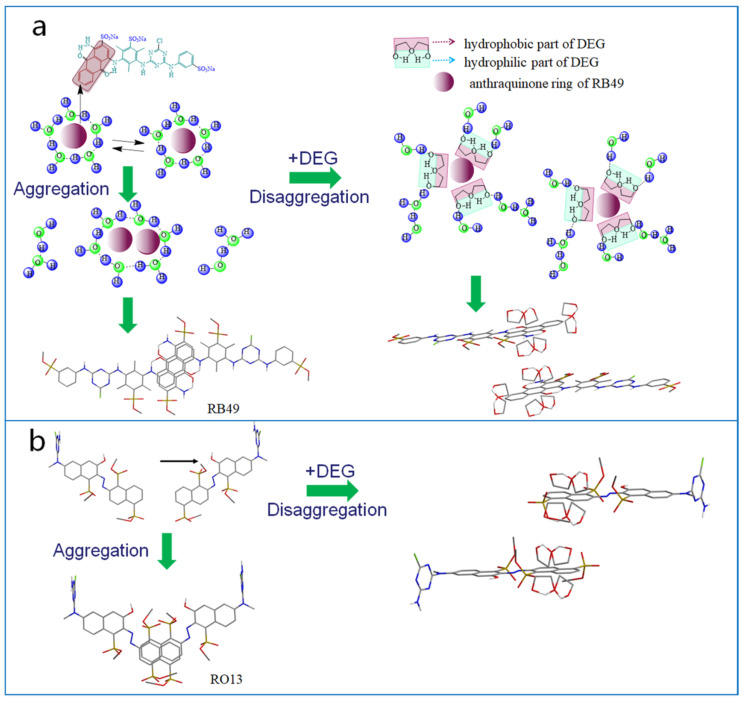
Schematic diagram of interaction between dye molecules and separation effect of DEG molecules to dye molecules: (**a**) Blue 49 and (**b**) Orange 13.

The possible interaction between DEG molecules and dye molecules is shown in Figure 3. It was confirmed that the hydrophobic parts of dye molecules are prone to aggregation to reduce the number of the water molecules at the hydration layer interface, release free water molecules, increase system entropy, and reduce surface free energy [36,37]. At high concentrations, dye molecules exist in the mixed form of monomers and aggregates. As shown in Figure 3a, in the absence of DEG, the hydrophobic anthraquinone rings of the Reactive Blue 49 tended to gather, leading to the formation of dye-molecule aggregations. After the addition of DEG, DEG molecules break the icelike-structure water around the anthraquinone ring of Blue 49, the anthraquinone rings were surrounded by the hydrophobic portion of DEG molecules, which made the anthraquinone ring hydrophilic. As a result, the anthraquinone ring could be stabilized in aqueous solution without forming aggregations. The phenomenon also happened to Orange 13 dyes. The addition of DEG resulted in the disaggregation of dye molecules (Figure 3b). While the hydrophobic area of the naphthalene ring of the Orange 13 dye molecules was smaller than that of the anthraquinone ring of Blue 49, fewer DEG molecules were required to disintegrate the naphthalene rings than to disintegrate the anthraquinone rings. Therefore, the disaggregation effect of DEG to Orange 13 was more obvious than with Blue 49 when the same amount of DEG was used.

### 2.2. Effect of DEG on Droplet Formation

The rheological properties and surface tension of the dye solutions have crucial influence on droplet formation. Very high surface tension can make it difficult for droplets to escape from the nozzle, and too low surface tension may result in droplet breakup [38,39,40,41,42].

**Figure 4:**
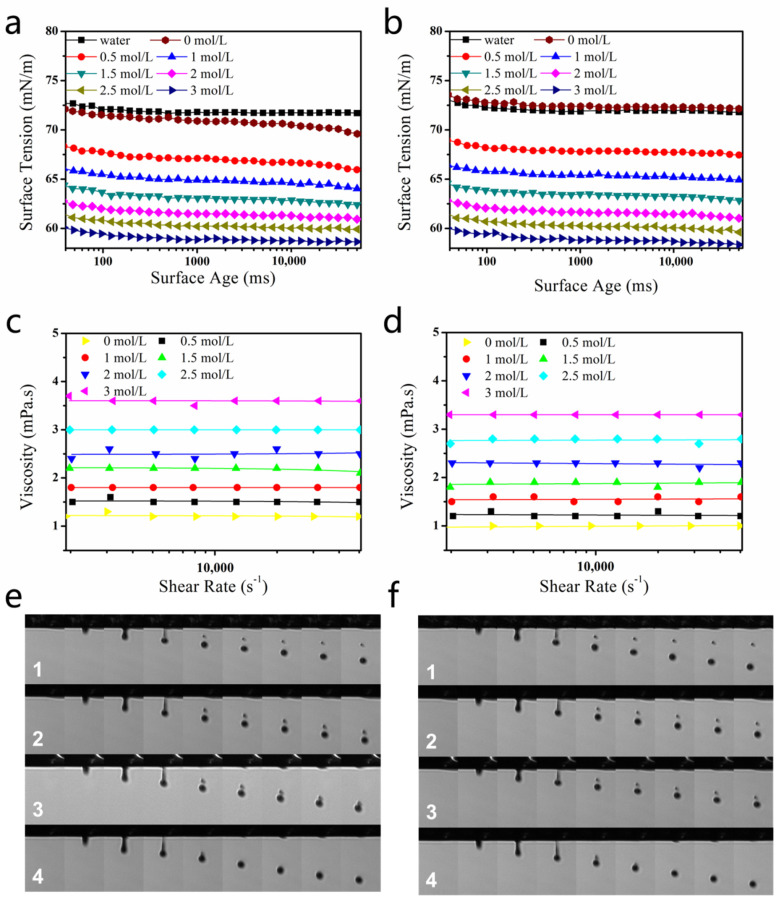
(**a**,**b**) Surface-tension values of 120 mmol/L dye solutions containing different amounts of DEG: (**a**) Reactive Blue 49 and (**b**) Reactive Orange 13. (**c**,**d**) Viscosity values of 120 mmol/L dye solutions with different amounts of DEG: (**c**) Blue 49 and (**d**) Orange 13. (**e**) Droplet formation of 120 mmol/L Reactive Blue 49 solution with different concentrations of DEG: (**1**) 0, (**2**) 1, (**3**) 2, and (**4**) 3 mol/L. (**f**) Droplet formation of 120 mmol/L Reactive Orange 13 dye solutions with different concentrations of DEG: (**1**) 0, (**2**) 1, (**3**) 2, and (**4**) 3 mol/L. Jetting frequency was 1000 Hz, temperature was 25 °C, and humidity was controlled in the range of 50–70%.

The surface tension of dye solutions (Orange 13 and Blue 49 molecules) with different concentrations of DEG is shown in Figure 4a,b. Both declined with the increase of DEG concentration.

**Figure 5:**
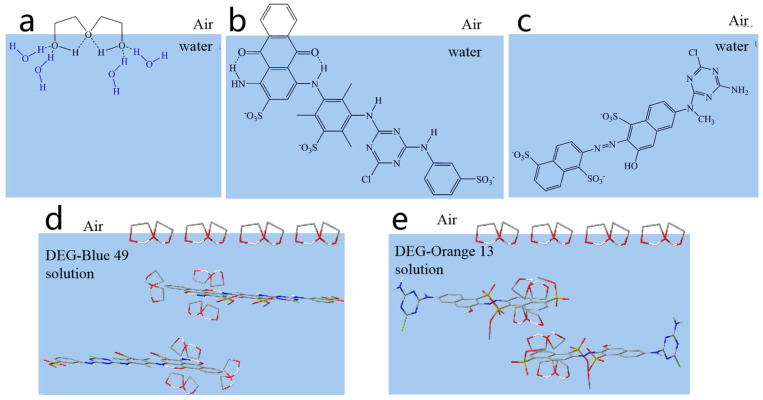
Arrangement of (**a**) DEG molecule, (**b**) Reactive Blue 49 molecule, and (**c**) Reactive Orange 13 molecule at air/water interface. (**d**,**e**) Arrangement of molecules at air/DEG –dye solution interface, (**d**) Reactive Blue 49-DEG solution, (**e**) Reactive Orange 13-DEG solution.

In aqueous solutions, water molecules around hydrophobic groups of molecules form an icelike structure [43,44]. Hydrophilic groups can be hydrated with water molecules or polar groups that can form hydrogen bonds with water molecules and break this structure [45]. To maintain the disorder of dye solution, the hydrophobic portions of molecules have to adsorb at the air–solution interface to release more free water molecules. The arrangement of DEG, Blue 49, and Orange 13 molecules at the air–water interface is shown in Figure 5. In the absence of DEG molecules, Blue 49 molecules can arrange on the surface of the air–water interface because there are not enough hydrophilic groups connecting to the anthraquinone group to completely break the icelike structure (Figure 5b), so the pure Blue 49 aqueous solution had lower surface tension than that of water. 

Orange 13 dyes were not able to arrange on the air/solution interface because they had sulfonic groups on the naphthalene rings (Figure 5c). As a result, they had almost identical surface-tension values with water. DEG had small molecules and a tendency of distributing on the air/solution interface, replacing the air–water interface with the air–DEG interface (Figure 5a). When DEG was added into the dye solutions, part of the DEG molecules were prone to interacting with the dye aggregations by hydrophobic interaction to break the icelike-structure water around the dye molecules, and part of the DEG molecules were arranged on the air–solution interface (Figure 5d,e). As a result, the surface tension of the dye solutions with DEG declined.

Figure 4c,d shows the viscosities of dye solutions. The viscosities of dye–DEG solutions did not change with increasing shear frequency, confirming that the solutions were Newtonian fluids. 

Because the relative molecular weight of Blue 49 (882) was larger than that of Orange 13 (762), and π–π interaction between the anthraquinone groups of Blue 49 was stronger than that between the naphthalene groups of Orange 13 [28], the pure Blue 49 solution had larger viscosity than that of the pure Orange 13 solution. In addition, the apparent viscosity of the dye solutions increased when larger concentrations of DEG were added into the solutions. Intermolecular forces such as van der Waals forces and hydrogen bonds are important in determining solution viscosity [46]. DEG had high viscosity, and DEG molecules could form numerous hydrogen bonds with water molecules. As a result, introducing DEG in the dye solution would accordingly enhance friction force and increase solution viscosity.

Figure 4e,f shows the droplet formation of the Blue 49 and Orange 13 dye solutions with different concentrations of DEG. For both dye solutions, the pure dye solution was easy to separate. As a result of low viscosity, surface tension broke the main droplet off from the neck of the droplet and applied downward acceleration to the main droplet. The receding filament formed a satellite droplet [40]. When DEG concentration was low, the surface tension of the dye solution was still too high; therefore, the main droplet dropped rapidly, and the satellite droplet could not catch up with it. With the increase of DEG concentration, the generated dye solution improved droplet quality. The satellite ink droplet of the Blue 49 solution almost merged with the main droplet when 2 mol/L DEG was added into the Blue 49 dye solution. When DEG concentration rose to 3 mol/L, there was no satellite ink droplet. Higher DEG concentration was required to completely eliminate the satellite ink droplet in Orange 13 dye solution than that required in the Blue 49 dye solution, probably because the former had slightly higher surface tension.

### 2.3. DEG Effect on Printing Performance

**Figure 6:**
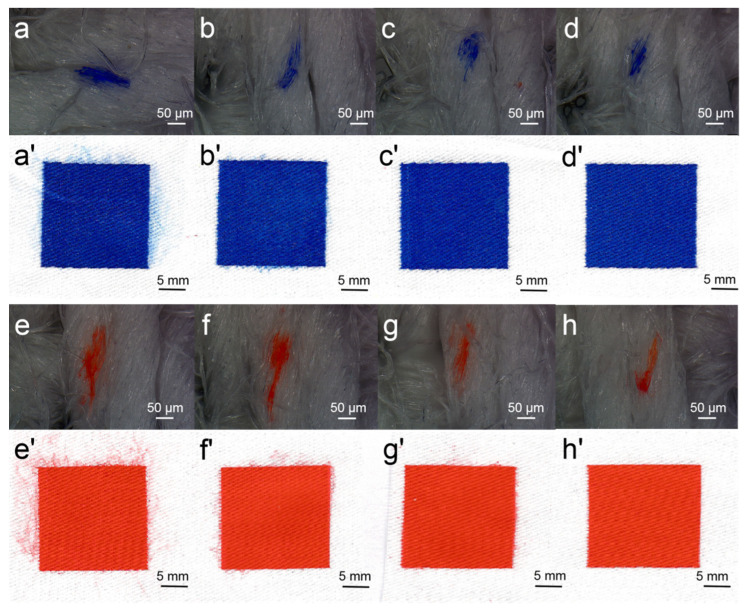
Ink-jet ink droplet spreading and printed images: (**a**–**d**) 120 mmol/L Reactive Blue 49 solutions with different concentrations of DEG: (**a**) 0, (**b**) 1, (**c**) 2, and (**d**) 3 mol/L. (**a’**–**d’**) Corresponding photos of cotton fabrics treated by Reactive Orange 13 from (**a**–**d**); (**e**,**f**) 120 mmol/L Reactive Orange 13 dye solutions with different concentrations of DEG (**e**,**f**): (**e**) 0, (**f**) 1, (**g**) 2, and (**h**) 3 mol/L; (**e’**–**h’**) corresponding photos of cotton fabrics treated by Reactive Blue 49 from (**e**,**f**).

A drop of ink was sprayed onto the cotton fabric to observe the droplet spreading area on the cotton fabric and printed images using the DEG–dye solution. The spreading of ink droplets and printed images is shown in Figure 6. The spreading area of the droplet apparently decreased as DEG concentration increased in the dye solutions of Blue 49 and Orange 13. This could be explained as the introduction of DEG improving the viscosity of the dye solution and increasing the inherent interaction force, which contributed to the prevention of ink spreading. The spreading area of the Orange 13 droplet was also larger than that of the Blue 49 solution when DEG concentration was the same. As the viscosity of Blue 49 ink was higher than that of the Orange 13 solution, the result also confirmed that the solution viscosity had a significant effect on hindering the ink droplet from excessively spreading on fabrics.

When DEG concentration was low, the printed images clearly showed that the ink droplet was apt to overspread and the image edge permeated, resulting in an obvious feather edge effect. In addition, the generated satellite ink droplet dispersed on the fabric made the boundary of printed color block unclear, showing reduced image resolution. In comparison with the Orange 13 dye solution, the Blue 49 solution had better pattern quality at the same concentration of DEG, which could be attributed to its high viscosity and smaller surface tension. When the concentration of DEG reached 2 mol/L, the image of the Blue 49 dye solution was satisfyingly clear; for the Orange 13 dye solution, higher DEG concentration was needed for high-resolution image printing.

## 3. Materials and Methods

### 3.1. Materials

Diethylene was purchased from Shanghai Macklin Biochemical Co., Ltd. (Shanghai, China). Pure C.I. Reactive Blue 49 and C.I. Reactive Orange 13 for ink preparation were purchased from Everlight Chemical Industrial Co., Ltd. (Taipei, China) without further purification. The chemical structures of Blue 49, Orange 13, and diethylene glycols are shown in Figure 1. Pure water with a conductivity of 0.9 µS/cm was used in all experiments. Cotton fabrics (32 × 21/133 × 60) were provided by YuYue Home Textiles Co., Ltd. (Binzhou, China)

### 3.2. Preparation of Reactive-Dye Solution

Dye solutions were prepared in a 100 mL volumetric flask. Certain amounts of dyes and DEG were accurately weighed on an AL104 precision electronic balance, and then put into a 100 mL beaker with 50 mL water. The weighted reactive dye was slowly dissolved in a mixture of water and DEG under stirring by a HJ-6A digital multihead magnetic stirrer (Shanghai Shuangjie Experimental Equipment Co., Ltd., Shanghai, China) at 25 °C for two hours. The prepared solution was transferred to a 100 mL volumetric flask; distilled water was used to wash the beaker three times, and then poured into the volumetric flask. DEG concentrations of in dye solutions were 0, 0.5, 1.0, 1.5, 2.0, 2.5, 3.0 mol/L, respectively, while the concentration of dyes were maintained at 120 mmol/L.

### 3.3. UV–Visible Absorption Spectroscopy

U-3900H ultraviolet spectrophotometer (Hitachi High-Tech Co., Ltd., Tokyo, Japan) was used to measure the visible absorption spectra of dye inks contained in a 0.01 mm thickness cuvette at 25 ℃.

### 3.4. Rheological-Property Test

The viscosity of Blue 49/Orange 13 and diethylene solutions was measured by a FLUDICAM RHEO microfluidic visual rheometer (Formulaction company, Toulouse, France) at 25 °C.

### 3.5. Surface-Tension Test

The surface tension of Blue 49/Orange 13 and diethylene solutions was measured by a SITA surface tension meter (SITA, Dresden, Germany) at 25 °C.

### 3.6. Ink-Jet-Printing and Droplet-Spreading Performance on Fabric

The droplet formation of all solutions was observed through a Jetlab II ink-jet printing system (Shanghai Ruidu Photoelectric Co., Ltd., Shanghai, China) that was equipped with a nMJ-AT printing head with the nozzle diameter of 30 μm. The inkjet printing was conducted at 25 °C with a droplet jet frequency of 1000 Hz, and the humidity was controlled in the range between 50–70%. The ink droplet spreading behavior of all solutions on the cotton fabrics was observed through a Leica DVM6M ultra depth microscope (Leica company, Wetzlar, Germany).

## 4. Conclusions

This study showed that reactive-dye chromophores are featured with aggregation morphology in the aqueous solution, and DEG molecules can disaggregate big dye aggregations to small aggregations. The disaggregation effect in the Orange 13 solution was more obvious than that in the Blue 49 solution at the same DEG concentration. The addition of DEG could reduce surface tension and increase the viscosity of dye solutions; as a result, DEG could improve the formation of ink droplets and prevent the formation of satellite droplets. When DEG was added in the dye solutions, they coulk hinder ink from percolating and printing color blocks with clear edges and high resolutions. It was also found that the Blue 49 solution had a more obvious decrease in surface tension and improvement in viscosity than those of the Orange 13 dye solution. As a result, the Blue 49 solution had fewer satellite droplets, a smaller spreading area on the fabric, and better printing performance than Orange 13 dye solution when the same concentration of DEG was used.
